# Plasma cell-free circRNAs panel act as fingerprint predicts the occurrence of laryngeal squamous cell carcinoma

**DOI:** 10.18632/aging.203215

**Published:** 2021-07-01

**Authors:** Jiahui Han, Qiuhong Lin, Chunguang Dong

**Affiliations:** 1 Department of Otolaryngology Head and Neck Surgery, The First People's Hospital of Lianyungang, The Affiliated Lianyungang Hospital of Xuzhou Medical University, Lianyungang, Jiangsu Province 222061, PR China

**Keywords:** plasma, circRNA, laryngeal squamous cell carcinoma, risk score function, receiver operating characteristic curve

## Abstract

Background: Circular RNAs (circRNAs) have recently emerged as a new class of RNAs, highly enriched in the human tissues and very stable within cells, exosomes and body fluids. In this study, we aimed to screen the plasma cell-free derived circRNAs in laryngeal squamous cell carcinoma (LSCC) and investigate whether these circRNAs could predicted LSCC as potential biomarkers.

Methods: The circRNA microarray was employed with three samples in each group to screen the dysregulated circRNAs isolated from plasma samples. The top 20 circRNAs were first selected as candidates with the upregulated level in the plasma of LSCC.

Results: Further validation found that only circ_0019201, circ_0011773 and circ_0122790 was consistent with training set. The ROC curve also revealed a high diagnostic ability an area under ROC curve value (AUC) for single circRNA and combined. The AUC for circ_0019201, circ_0011773 and circ_0122790 and the combined was 0.933, 0.908, 0.965 and 0.990 in training set. For the validation set, the AUC was 0.766, 0.864, 0.908 and 0.951. The three circRNAs were further investigated with stable expression in human plasma samples.

Conclusions: The plasma derived circ_0019201, circ_0011773 and circ_0122790 might be the potential biomarker for predicting the LSCC.

## INTRODUCTION

Laryngeal squamous cell carcinoma (LSCC) is one of the most common tumor pathologic types of the head and neck [1, 2]. According to the results of current epidemiological studies, smoking, HPV infection, laryngeal reflux and environmental occupational exposure are considered to be high risk factors for laryngeal cancer. [3, 4]. However, the molecular mechanism of the pathogenesis of LSCC remains unclear. In addition, although efforts have been made to develop an effective treatment for LSCC, the mortality rate for patients with LSCC remains high, with an overall 5-year survival rate of less than 50% [5]. With the development of generation sequencing technology, many researchers have used omics data to predict the occurrence or prognosis of tumors. Studies have shown that the up-regulation and down-regulation of specific gene expression in serum may be associated with clear diagnosis and prognosis of tumor [6, 7]. In clinical practice, squamous cell carcinoma of the neck is often diagnosed as other neck diseases, and in many cases, delayed diagnosis and treatment are inevitable. Therefore, it is of great clinical significance to develop a specific biomarker for cervical squamous cell carcinoma.

With the development of high-throughput sequencing technology, more and more non-coding RNA (ncRNA) was found in human genome. An increasing number of evidences show that non-coding RNAs can participate in cell biological functions through various promotion and play important regulatory roles [8]. Circular RNAs (circRNAs) are newly identified members of the non-coding RNA family, and they are also the focus of current research in non-coding RNA. Structurally, circRNAs are single-stranded, closed-loop structures that lack a 5′-ended cap or a 3′-ended poly(A) tail and are formed by covalent bonding with each other [9, 10]. Furthermore, researchers have indicated the differential circRNA profiles of the serum exosomes derived from patients with other human malignant tumors, such as gastric cancer, colon cancer and hepatocellular carcinoma [11, 12]. However, no evidence was found regarding the circRNA in LSCC patients.

In this study, we investigated the function of circulating cell-free circRNAs in plasma as biomarkers for LSCC. First, we integrated abnormal/deregulated circRNAs by analyzing the microarray data from the plasma samples of LSCC patients and non-cancer controls. The top 20 dysregulated circRNAs were then confirmed and validated by reverse transcriptional quantitative polymerase chain reaction (RT-qPCR) assay. A multi-stage validation, including a training group and a validation group set, was then performed to test whether abnormally expressed plasma derived circRNAs which might predict LSCC in healthy volunteers.

## RESULTS

### The circRNA expression profile in LSCC and control group

**Figure 1:**
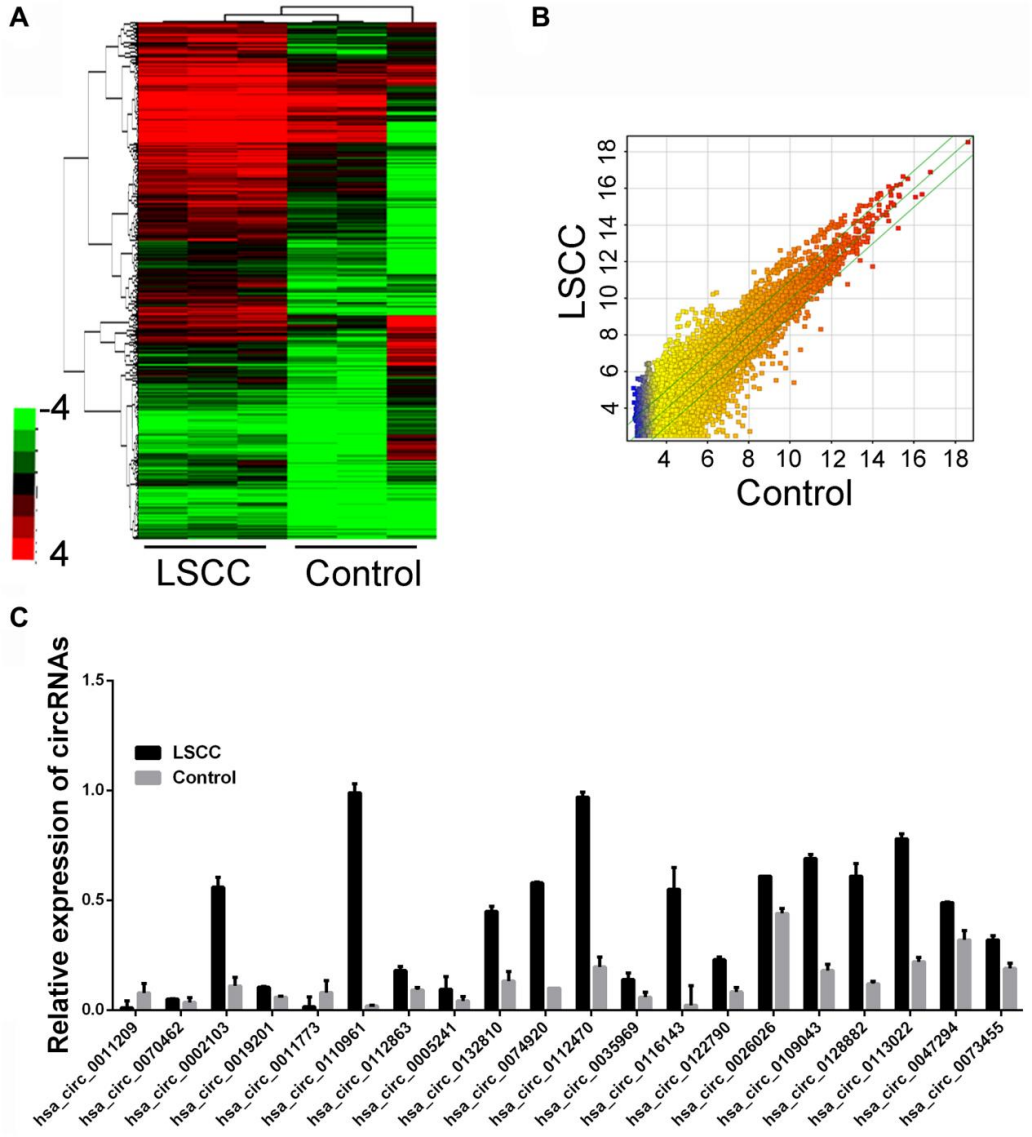
**circRNA expression profiling in circulating samples and tissues samples.** (**A**) Cluster analysis and scatter plot of the different expressed circRNAs (Three plasma samples from patients diagnosed with LSCC and three cancer-free controls). (**B**) Rhe scatter plots of dysregulated circRNA, *p* < 0.05. (**C**) The top upregulated circRNAs in LSCC group through microarray detection.

The analysis of circRNA microarray indicted that the cluster diagram showed the circRNA lineages with abnormal differentially expressed circRNAs among each group, while the circRNAs with statistically significant differences among each group were presented by the volcano diagram (Figure 1A). Due to the spatio-temporal characteristics of circRNA expression, we set a specific threshold to ensure the feasibility of subsequent validation. The expression level of circRNA in the chip must meet a clear statistical significance (*P* < 0.05), and the detectable rate in the chip must be greater than 75%. Under the limitation of the above criteria, we found that compared with the control group, 122 circRNAs transcriptome specificity was increased in the LSCC group, and 98 circRNAs were confirmed to be down-regulated (Figure 1B). In order to reveal the potential biomarker for LSCC, we selected the top 20 increased circRNAs in LSCC group as candidate diagnostic makers (Figure 1C).

### Validation of significantly dysregulated circRNAs in plasma

Two staged validation of candidate circRNAs was used including 20 paired samples as training set and 100 paired samples as validation set. The detailed information of patients and healthy controls was summarized in Table 1. A randomly selected 20 paired LSCC and control samples was used. The top 20 circRNAs was first validated. Among the 20 candidate circRNAs, 10 circRNAs was confirmed with significant different expression; however, 5 circRNAs was consistent with microarray results while the rest circRNAs was presented with decreased level in LSCC group.

**Table 1:** Clinicopathological characteristics of LSCC and cancer-free control samples.

**Characteristics**	**LSCC**	**Control**	***P* value**
**N**	120	120	
**Age Mean (SE) year**	55.12 (4.31)	56.25 (5.77)	0.21^a^
**Gender (male/female)**	79/41	82/38	0.68^b^
Location			
Lower	21		
Middle	68		
Upper	31		
**Tumor size**			
<4cm	55		
≥4cm	65		
**TNM stage (I: II: III)**	62:33:25		
**Lymph node metastasis**			
Yes	89		
No	31		

^a^Student *t*-test.

^b^Chi-square test.

Based on the results in training set, the rest 100 paired samples were enrolled as validation set. We next examine the expression of the five candidate circRNAs in validation set. We found that hsa_circ_0055202, hsa_circ_0074920 and hsa_circ_0043722 was confirmed with higher expression level in LSCC, while hsa_circ_0010178 presented no significant. The hsa_circ_0009760 presented a higher expression in LSCC; however, the *p* value was <0.05.

**Figure 2:**
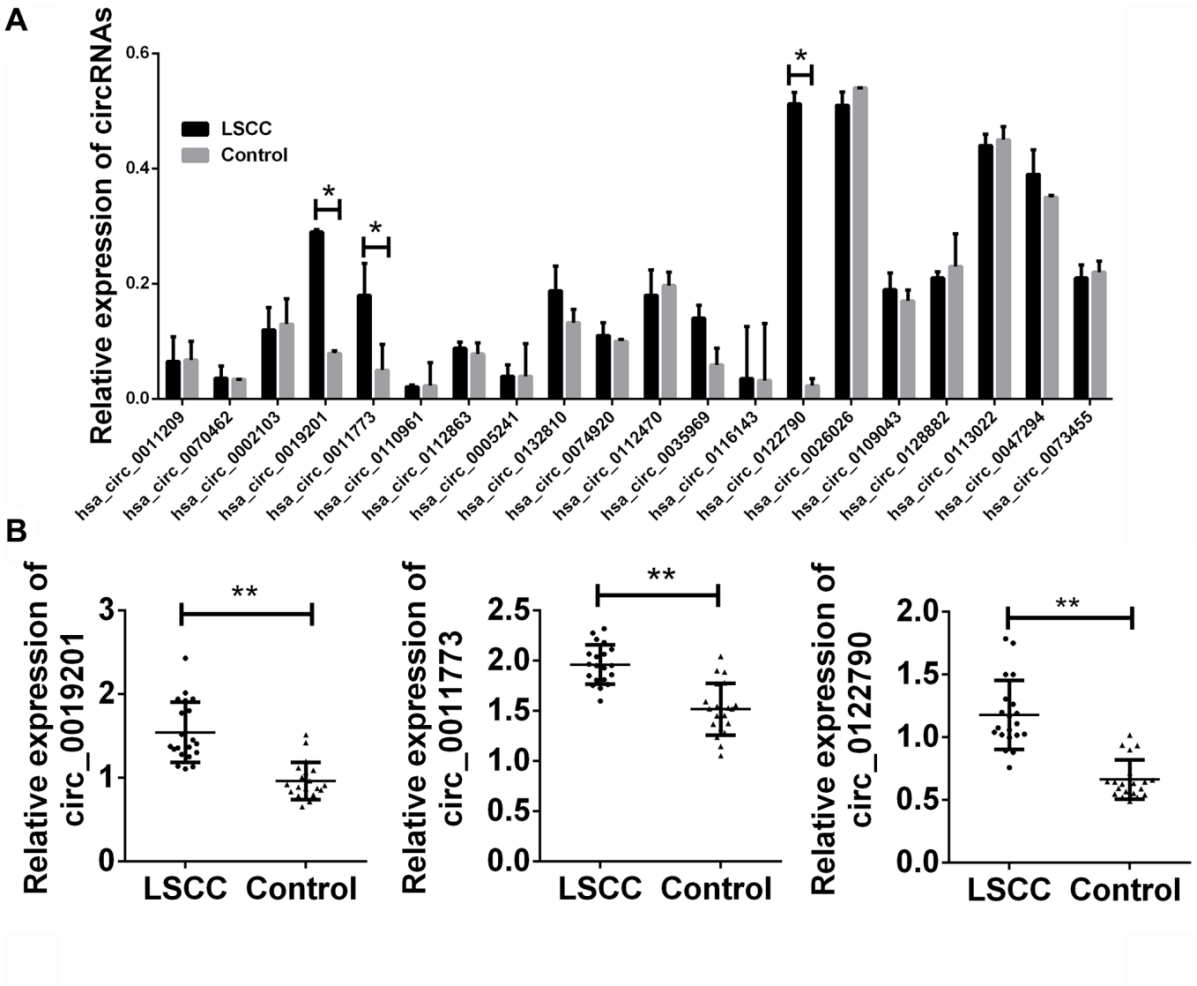
**Validation of candidate circRNA in training set.** (**A**) Total 20 paired plasma from LSCC patients, and 20 cancer-free controls were used in RT-qPCR analysis. (**B**) The detailed expression of circ_0019201, circ_0011773 and circ_0122790 in LSCC and control groups. Data was presented as mean ± SEM. Data was analyzed with student *t* test. n.s. indicated no significant, ^*^ indicated *p* < 0.05 and ^**^ indicated *p* < 0.01.

Two-stage validation of candidate circRNAs was performed, with 20 pairs of paired samples as the training set and 100 pairs as the validation set. As shown in Figure 2A, 20 pairs of LSCC and control samples were randomly selected. The top 20 circRNAs were identified for the first time. Among the 20 candidate circRNAs, three circRNAs entitled circ_0019201, circ_0011773 and circ_0122790 were proved to be differentially expressed and consistent with the microarray results. However, another four circRNAs were inconsistent with the microarray results, and the remaining circRNAs presented no difference between LSCC group and control group (Figure 2B).

**Figure 3:**
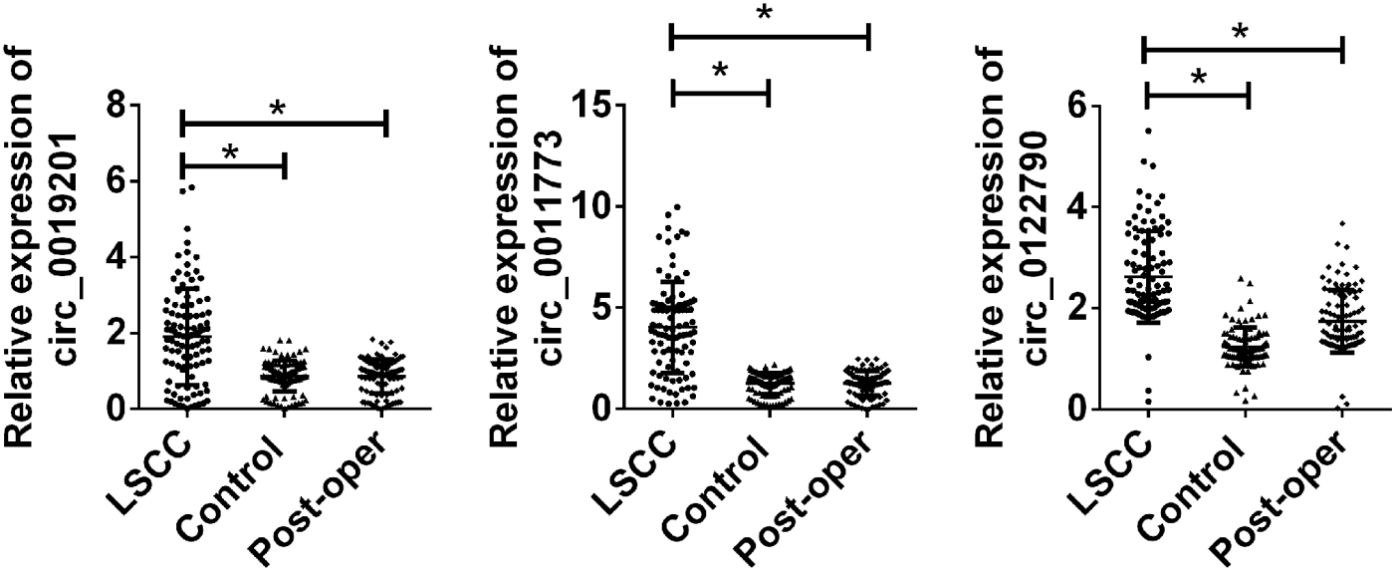
**Validation of candidate circRNA in n validation set.** Total 100 paired plasma from LSCC patients, and 100 cancer-free controls were used in RT-qPCR analysis. Data was presented as mean ± SEM. Data was analyzed with student *t* test, ^*^ indicated *p* < 0.05.

Based on the results of the training set, the remaining 100 paired samples were enrolled in the verification set. We then studied the expression of the circRNAs validation set of the three candidates. As shown in Figure 3, we found that circ_0019201, circ_0011773 and circ_0122790 had higher expression levels confirmed by LSCC. Furthermore, the expression of the three circRNAs presented a remarkable decreasing level after surgical excision.

### Risk score analysis

**Figure 4:**
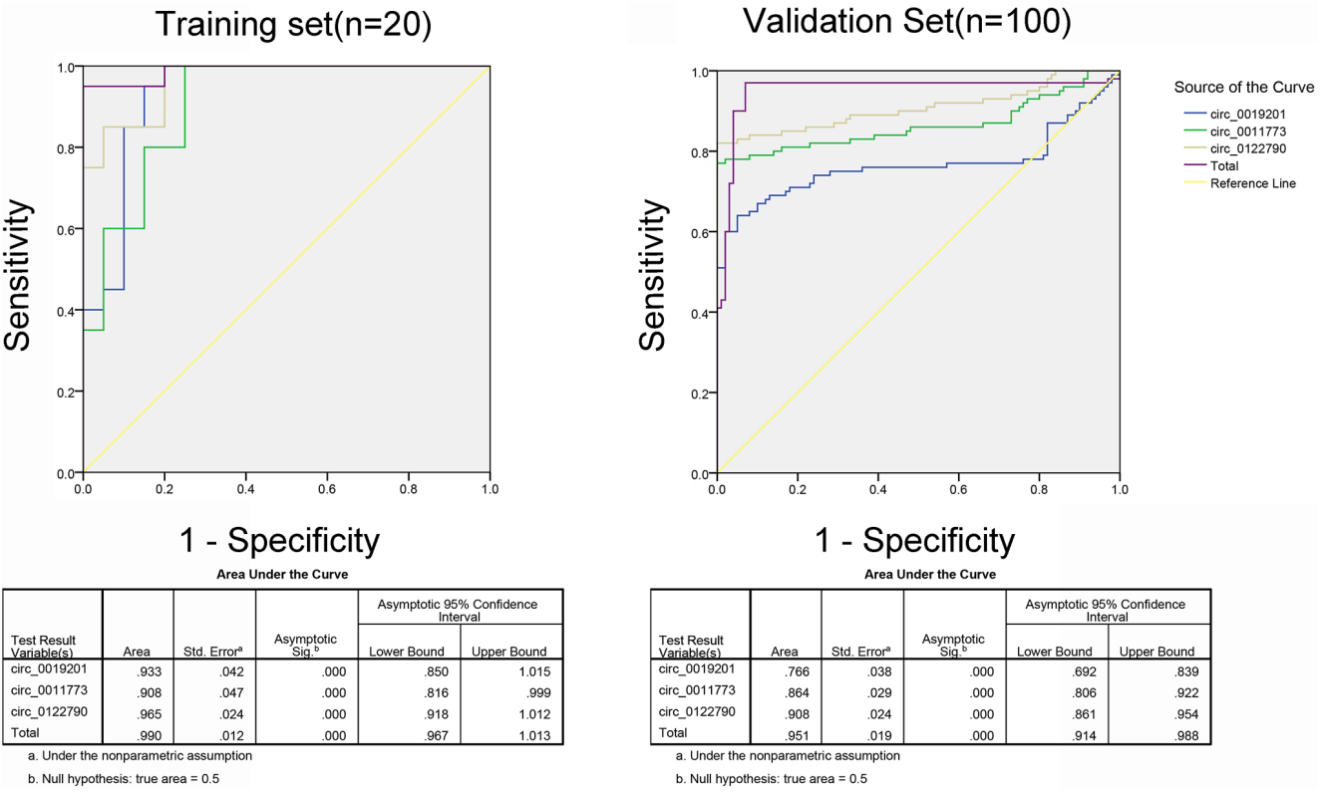
ROC analysis of the three potential biomarkers for LSCC by using risk score analysis.

To further explore the accuracy and specificity of these three circRNAs as potential features, we used a risk scoring formula to evaluate the diagnostic value of these three circRNAs. Firstly, we divided the control and case groups in the training set according to the 95% confidence interval (95% CI) for the control group. Logistic regression analysis was used to calculate the risk score. All plasma samples were then divided into a high-risk group (possibly LSCC) and a low-risk group (expected to be a control group). We defined the threshold as the maximum of sensitivity plus specificity. The positive predictive value (PPV) and negative predictive value (NPV) calculated in the training set were 90% and 85%, respectively. We further applied the same values to calculate risk scores for the validation set sample, with PPV and NPV of 90% and 89%, respectively (Table 2). In addition, we also used ROC curve analysis to evaluate the predictive diagnostic value of circRNAs for LSCC. The areas under the ROC curve of the three circRNAs were 0.933, 0.908 and 0.965, respectively, which could well distinguish the areas under the ROC curve between the LSCC patients and the control group. In the validation set of amplified samples, the areas under the ROC curve of the three circRNAs were 0.766, 0.864 and 0.908, respectively. Combined with these three circRNAs, the area under the ROC curve between LSCC patients and the control group was 0.951 (Figure 4).

**Table 2:** Risk score analysis of in LSCC and cancer-free control plasma samples.

**Score**	**0–7.08**	**7.08–11.02**	**PPV^a^**	**NPV^b^**
**Training set**			0.90	0.85
LSCC	2	18		
Control	17	3		
**Validation set**			0.90	0.89
LSCC	10	90		
Control	89	11		

^a^PPV, positive predictive value.

^b^NPV, negative predictive value.

### Stability detection of circRNAs in plasma samples

**Figure 5:**
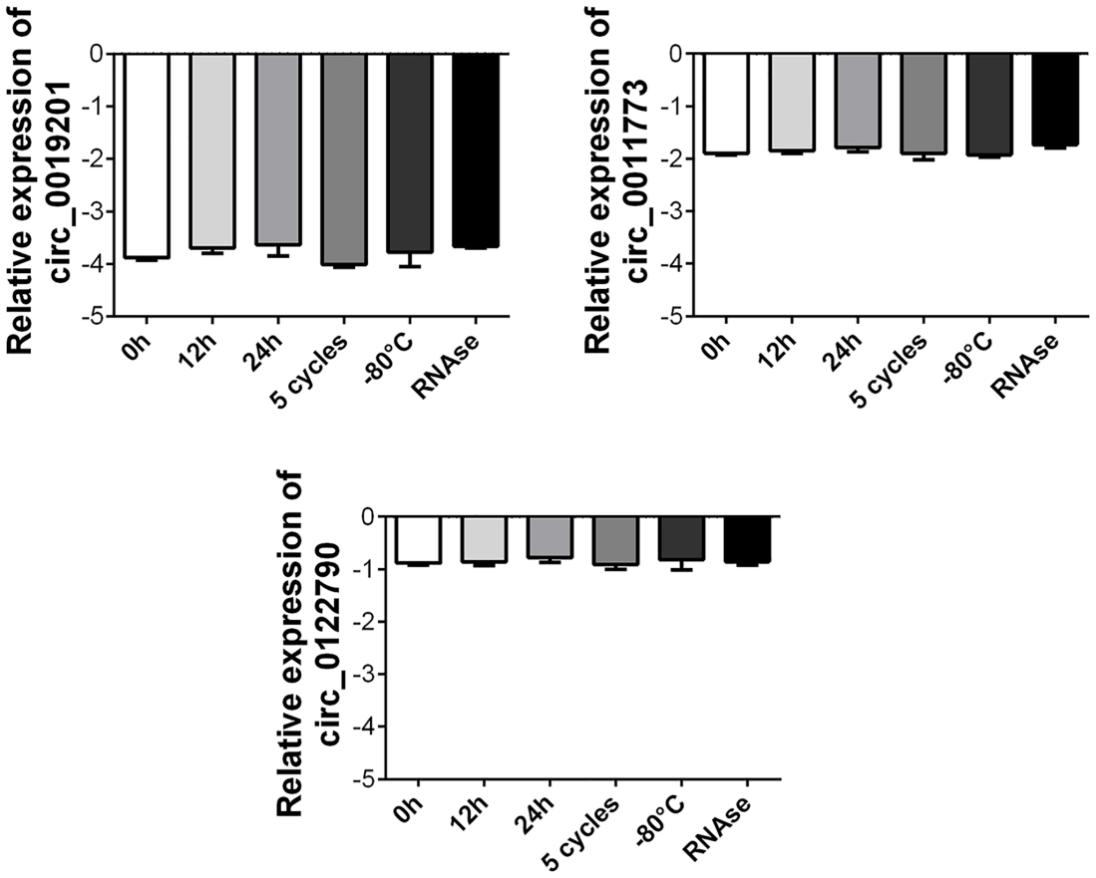
**Stability detection of the three circRNAs in human plasma.** RT-qPCR was applied for detecting the expression level of the three circRNAs. Data was presented as mean ± SEM with log-transformed. No significant difference was observed in each group.

Next, we amplified these three circRNAs in five healthy controls. Human plasma obtained from three healthy controls was frozen and thawed for 5 cycles at room temperature for 0, 12, and 24 h. It was stored at –80°C for approximately seven days and digested with RNase. We found that the expression levels of these three circRNAs did not change, indicating that circ_0019201, circ_0011773, and circ_0122790 were stably expressed and detectable in human plasma (Figure 5).

## DISCUSSION

In the past decade, great progress has been made in the research and treatment of LSCC [13, 14]. However, treatment resistance increases the recurrence rate of LSCC and patients who undergo total laryngectomy have a poor quality of life [15]. This study screened the differentially expressed genes to provide ideas for subsequent studies, including the exploration of potential diagnostic targets, which is conducive to the risk assessment of LSCC at an earlier stage.

Previous published studies on biomarkers of LSCC were mainly focused on proteins and miRNAs or lncRNAs. For example, some researchers have found that HOTAIR, a long non-coding RNA, was significantly increased in LSCC and is closely related to tumor progression, as well as the occurrence, metastasis, and poor prognosis of tumors [16]. In addition, abnormally elevated MALAT1 expression was also found in LSCC tumor tissues, suggesting a poor prognosis for patients [17]. The long non-coding RNA UCA1 was also highly expressed in LSCC [18]. However, the current studies on non-coding RNA in LSCC mainly focus on tumor tissues and tumor cell models, and there were still few studies on the exploration of LSCC-related diagnostic markers in cell-free plasma samples. Therefore, starting from the plasma samples of LSCC patients, this study focused on cell-free circRNAs in plasma samples by using high-throughput microarray technology. Based on the stability of circRNA expression, the potential of circRNA as a disease risk prediction target was further explored, and the study was innovative to a certain extent.

Circulating non-coding RNAs have recently emerged as novel biomarkers for cancer development and progression. Among the studies on non-coding RNAs as diagnostic markers, miRNAs were the first to be reported, and there have been commercialized kits for the diagnosis of disease models, followed by long non-coding RNAs. However, with the deepening understanding of non-coding RNA, more and more attention has been paid to the clinical value of circRNAs in tumor diagnosis. Xu et al. identified circRNA_0000826 as a potential diagnostic biomarker for liver metastasis from colorectal cancer by RNA sequencing [19]. In addition, researcher also found that circ-kiaa1244, derived from gastric cancer tissue, could serve as a novel circulating biomarker for gastric cancer detection [11]. These results all proved the feasibility of peripheral circulating circRNA as a future diagnostic target in LSCC.

In summary, in this study, we used a microarray-based approach to screen the potential fingerprints of LSCC. we found that the circ_0019201, circ_0011773 and circ_0122790 Panel may be able to predict LSCC in the normal population with relatively high sensitivity and specificity, suggesting that circ_0019201, circ_0011773 and circ_0122790 may be used as an early disease prediction model for LSCC in the future. However, due to the limitation of the sample size in this study, more samples are needed for verification, and further studies are needed to confirm the potential regulatory mechanism of these circRNAs in the development of LSCC.

## MATERIALS AND METHODS

### Study design

A retrospective case-control study was conducted at the First People's Hospital of Lianyungang. A total of 120 patients with LSCC who were diagnosed with LSCC in department of Otolaryngology Head and Neck Surgery of the First People's Hospital of Lianyungang was enrolled from January 1, 2015, to July 1, 2019. This study was approved by an institutional review board of The First People's Hospital of Lianyungang. Written informed consents were obtained from all enrolled subjects. Blood samples from all patients were taken preoperatively or before chemoradiotherapy. Peripheral blood samples were obtained and stored in EDTA anticoagulant tubes. The blood samples were first pretreated by centrifugation at 3000rpm for 10 min. Then the plasma and precipitated samples were separated and stored in a –80°C refrigerator. All patients signed informed consent before receiving specimens. All patients with LSCC were identified by postoperative histopathological diagnosis. The American Joint Committee on Cancer (AJCC) 10th Edition Tumor-Lymph Node Metastasis (TNM) system was used for staging diagnosis in combination with preoperative imaging examination. All studies were conducted in accordance with the Helsinki Declaration.

### circRNA microarray and analysis

RNA was extracted from three plasma samples diagnosed with LSCC and three healthy controls as circulating samples, both of which were used for microarray detection. Each sample was tested with a 1.0 μg total RNA. The microarray was detected by using Human CircRNA Microarray V2 (CapitalBio, Beijing, China). We screened out all the low-expression genes before performing other analyses. We only retained genes with at least half markers in five out of samples. This reduces the initial 37,681 input genes in each sample to about 15,000 detected genes. In the following analysis, we used this set of expression filter genes. We performed a bilateral Mann-Whitney *U* test performed by Wilcox. Finally, we use the Benjamin-Hochberg correction (R. P. Adjust function) to explain the multiple tests. For the purpose of analysis, we found that the genes significantly differentially expressed were those with a Benjamin-Hochberg *Q* value <0.05. We also used the Limma package to perform parallel differential expression analysis. All further data analysis and visualization is performed using custom R scripts.

### RNA extraction and quantitative real-time PCR (qRT-PCR)

RNA was extracted from plasma samples using the Trizol method. The extracted RNA was qualitatively and quantitatively tested to ensure that it met subsequent validation. RNA integrity was assessed by standard denatured 1% agarose gel electrophoresis. In this study, nematode miRNA (cell-miR-39, Applied Biosystems, Foster City, CA, USA) was added to each sample as an external reference. The cDNA was synthesized by Superscript First-Strand Synthesis System (Invitrogen, Carlsbad, CA, USA). The PCR was conducted by using ABI 7900HT (Applied Biosystems, Foster City, CA, USA). The relative expression of circRNA was normalized to GAPDH and cell-miR-39 and calculated using 2^-ΔΔCQ^ method.

### Screening phase

The screening stage is mainly divided into two stages: test combination validation group. During the trial, 20 samples from each group of patients with LSCC were randomly included for data analysis along with 20 matched healthy controls. A total of 100 samples were randomly included in the validation group for analysis.

We used a risk scoring system to predict the predictive power of single circRNAs and multiple circRNAs as the most diagnostic markers. In the cases of high expression index, we preferred to the control group on the index's 95% reference range (95% CI) as a cut-off point, if a sample of the index's expression level is higher than the cut-off point, that is defined as 1, suggest it may be closer to case group, defined as 0, conversely said tend to be more with the control group. Logistic regression analysis was conducted with the real attributes of the samples to obtain the regression coefficient. The risk score was defined based on a linear combination of the expression levels of each circRNA. The regression coefficient calculated by the risk score of multiple circRNAs for the weight of use was derived from univariate logistic regression analysis for each circRNAs. The samples were sorted according to RSF and then divided into high risk groups (representing the LSCC group) and low risk groups (representing the predictive control individuals).

### Statistical analysis

If there is no special annotation, the results in this study were presented by Mean ± SEM. For continuous variables, we used ANOVA for analysis. Chi-square test was used for statistical analysis of the classification variables. All statistical analyses were performed using Stata10, and all charts were produced using GraphPad Prism.

### Availability of data and materials

The datasets used and/or analyzed during the current study are available from the corresponding author on reasonable request.

### Ethics approval and consent to participate

Ethical approval was obtained from the Ethics Committee of the First People's Hospital of Lianyungang. All procedures performed in studies involving human participants were in accordance with the ethical standards of the institutional and national research committee. Informed consent was obtained from all individual participants included in the study.
